# Leaf phenotypic variation and its response to environmental factors in natural populations of *Eucommia ulmoides*

**DOI:** 10.1186/s12870-023-04583-3

**Published:** 2023-11-15

**Authors:** Huimin Gong, Min Yang, Chaochun Wang, Chunlian Tian

**Affiliations:** 1 Key Laboratory of Hunan Forest Products and Chemical Industry Engineering, National and Local United Engineering Laboratory of Integrative Utilization Technology of Eucommia ulmoides, Jishou University, Zhangjiajie, 427000 China; 2 College of Biology and Environmental Sciences, Jishou University, Jishou, 416000 China

**Keywords:** *Eucommia ulmoides*, Leaf traits, Altitude, Climate factors, Correlation analysis

## Abstract

**Background:**

*Eucommia ulmoides* leaves have high medicinal and economic value as a dual-purpose substance for medicine and food. Employing leaves from 13 natural populations of *Eucommia ulmoides* as research objects, this study reveals the variation patterns of intra-specific and inter-specific trait variation and explores the response of leaf characteristics to geographical and climatic changes, aiming to provide a scientific basis for the efficient utilization of leaf resources and the breeding of superior varieties.

**Results:**

Descriptive statistical analysis and nested analysis of variance showed significant differences in 11 leaf traits of *Eucommia ulmoides* inter-populations and intra-populations, with an average coefficient of variation of 17.45%. The coefficient of variation for average leaf phenotypic traits is 20.77%, and the leaf phenotypic variation is mainly from the variation intra-populations. Principal component analysis reveals that the cumulative contribution rate of the top three principal components which mainly contributed to the phenotypic variation of *Eucommia ulmoides* leaves reached 74.98%, which could be sorted into size traits (34.57%), color traits (25.82%) and shape traits (14.58%). In addition, correlation analysis expresses there is a specific co-variation pattern among leaf traits, with a strong connection between shape, size, and color traits. Geographic and climatic distances are significantly correlated, and mantel test and correlation analysis indicate that leaf traits of *Eucommia ulmoides* are mainly influenced by altitude. With the increase of altitude, the leaves become smaller. Partial correlation analysis shows that after controlling climate factors, the correlation between some characters and geographical factors disappears significantly. Temperature and precipitation have a great influence on the variation of leaf phenotypic traits, and the larger the leaves are in areas with high temperature and heavy rainfall.

**Conclusions:**

These findings contribute to a further understanding of the leaf morphological characteristics of *Eucommia ulmoides* and the extent to which the environment influences leaf trait variation. They can provide a scientific basis for the protection and application of *Eucommia ulmoides* leaf resources in the future.

## Background

Plant phenotypic traits not only encompass morphological features such as those of leaves, fruits, and seeds but also serve as a direct indicator of genetic variation in plants [1, 2]. Leaves act as vital gateways for water and gas exchange between plants and the external environment. They constitute the primary organs for photosynthesis and transpiration, exerting significant influence on ecological material production, global carbon cycling, and water cycling [3]. Leaf phenotypic traits represent the most intuitive classification characteristics in plant taxonomy, also reflecting a plant’s adaptability to changing growth environments [4]. Variations in leaf morphology directly impact the physiological and biochemical processes in plants, closely correlating with a plant’s efficiency in acquiring and utilizing resources [5]. For instance, elliptical leaves exhibit higher photosynthetic and water use efficiency than lanceolate leaves [6, 7]. Additionally, leaf size can indicate the content of active components within the leaf [8, 9]. Variations in leaf phenotypes not only serve as a crucial metric for gauging genotypic variations but also reveal the patterns and underlying mechanisms of this variation. This lays a foundation for the genetic improvement, introduction, and domestication of plants [10].

Variation in leaf phenotypes is a manifestation of plants adapting to different habitats under selection pressures, reflecting the survival strategies evolved by plants in response to changing environments [11]. In recent years, with increasing attention to global climate change, research on plant leaf traits in response to the environment has gradually expanded [12, 13]. Studies have shown that factors such as temperature, light intensity, precipitation, latitude, longitude, altitude, and soil type have a significant impact on leaf traits [14, 15]. For instance, in regions with warm temperatures, abundant precipitation, and no direct strong sunlight, leaves tend to be larger. Conversely, in cold regions with strong sunlight or in dry or nutrient-poor soils, leaves tend to be smaller to avoid overheating or reduce water loss [16–18]. Specific leaf dry weight can reflect the adaptive characteristics of plants in different habitats [19]. Harsh environmental conditions and increasing altitude lead to an increase in specific leaf dry weight, which is detrimental to the growth of plants or communities [20]. To date, research on leaf phenotypic traits has overlooked the importance of intra-species trait variation in community dynamics. The impact of intra-species trait variation on ecosystem function is significant and should not be underestimated [13]. Therefore, conducting a quantitative study on intra-species trait variation along environmental gradients can support the prediction of species responses to climate change and the influence of the environment on trait variation. This has important implications for the breeding of superior plant varieties, conservation of endangered plant resources, and protection of biodiversity [21].

*Eucommia ulmoides* (*E. ulmoides*) is a rare and endangered species endemic to China. Due to the presence of various active components in its leaves, bark, and gum, it has become an important economic and industrial raw material tree species in China [22]. The central production areas are located in northwestern Hunan, northern Guizhou, western Guizhou, and northwestern Hubei, among others, covering a wide range of environmental conditions [23]. In response to future climate change, the overall distribution area is shifting towards the northwest and higher latitudes [24]. Therefore, studying the intraspecific variation of *Eucommia ulmoides* leaves (EULs) can provide deeper insights into its phenotypic differentiation and ecological adaptation. However, current research on EULs mainly focuses on chemical composition [25, 26], pharmacological uses [27, 28], potential distribution predictions [29], and whole-genome association analysis [30], with very little attention to the phenotypic trait variation of EULs. Meng et al. found that 14 leaf phenotypic traits from the *Eucommia* germplasm resource database showed rich variation (4.57-20.68%) [31]. Wang et al., on the other hand, found higher levels of variation in five leaf phenotypic traits within natural *Eucommia* populations (20.96-49.00%). In addition, a correlation analysis between leaf phenotypic traits and three climatic factors revealed that annual average temperature and precipitation significantly influenced leaf size [32]. Furthermore, due to the similarity in active components and pharmacological effects between *Eucommia* leaves and bark, EULs have a wide range of applications [33]. Previous studies have found that the content of isoquercitrin is closely related to leaf length, leaf area, and leaf perimeter [34]. This provides a scientific basis for further research on leaf phenotypic variation and its response to the environment in natural *Eucommia* populations with richer phenotypic variation.

In this study, climate and geographic data were collected from 13 natural populations in the central production area of *E. ulmoides*, and 11 leaf phenotypic traits were measured for 134 trees. The aim was to investigate the variation in leaf traits and its mechanisms of adaptation to geographic and climatic factors. This study aims to reveal the patterns of variation in leaf phenotypic traits, providing a scientific basis for the selection of superior *Eucommia* resources, efficient utilization of leaf resources, and practical production.

## Results

### Leaf morphological variation

**Fig. 1:**
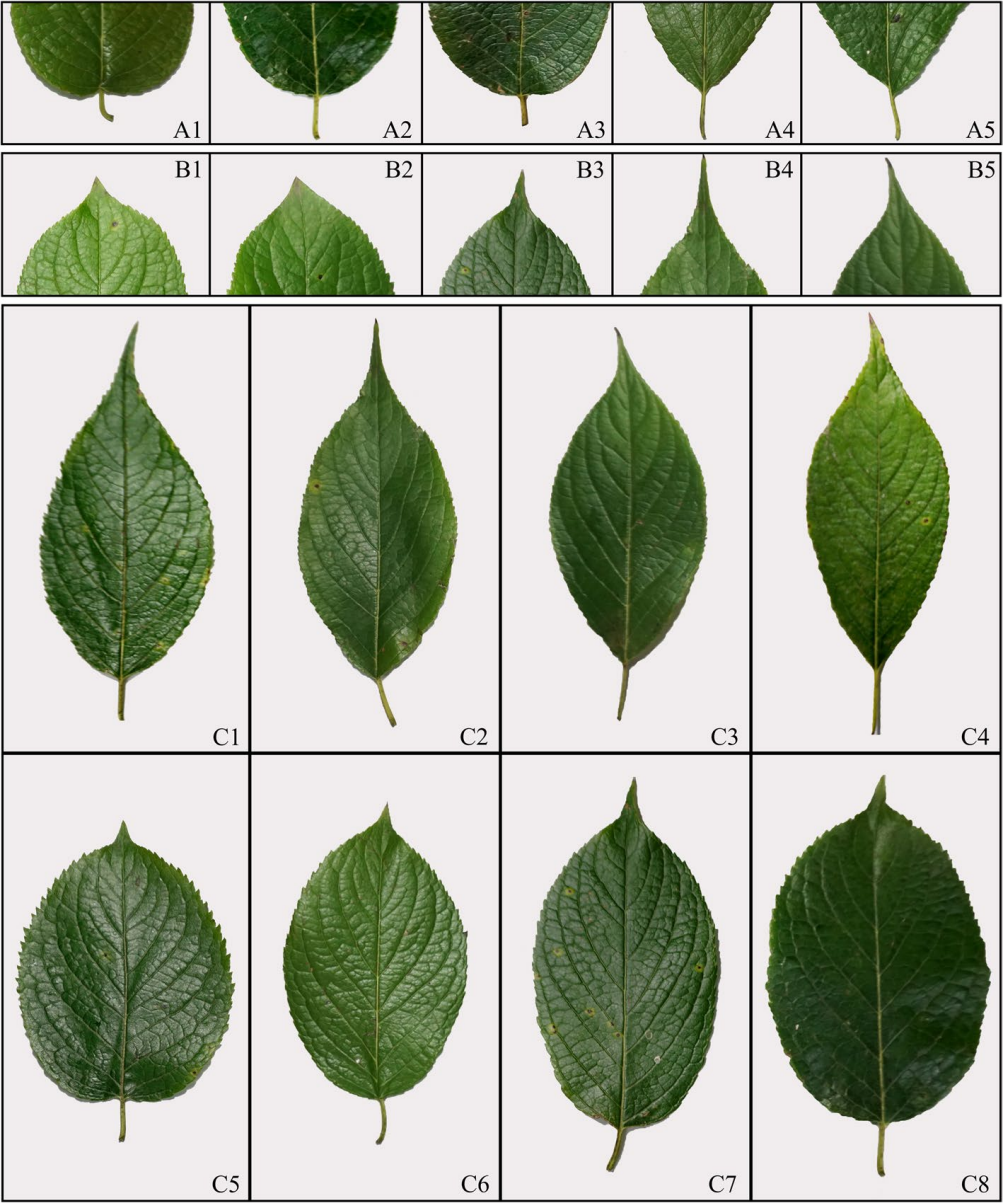
Variation types of *Eucommia ulmoides* leaf apex, leaf base, and leaf shape

 Scanning and morphological comparisons were performed on the leaf base, leaf tip, and leaf shape of 134 EULs. The results indicate that there is abundant variation in EULs (Fig. 1). The leaf base has five shapes: heart shape, round shape, truncate shape, cuneate shape and oblique shape (A1-A5). The leaf apex has five types: cuspidate, acute, acuminate, aristate and caudate (B1-B5). The leaf shape has eight types: lanceolate, ovate-lanceolate, ovate-oblanceolate, oblanceolate, round, ovate, oblong, and elliptical (C1-C8).

### Leaf phenotypic variation

Descriptive statistical analysis was conducted on leaf traits (Table 1), and the results indicated that all measured traits exhibited varying degrees of variation. The coefficient of variation ranged from 9.00 to 30.88%, with an average of 17.45%. Among them, the chlorophyll reference value (CRV) showed the highest coefficient of variation (30.88%), followed by leaf area (LA) (26.73%) and specific leaf dry weight (SLDW) (20.94%). The lowest coefficient of variation was observed for the blue component of leaves (BC) (9.00%). The coefficient of variation for the 3 leaf colour traits, the red component of leaves (RC) (11.64%), BC (9.00%), and green component of leaves (GC) (13.89%) was lower than that for the other 8 leaf traits.

**Table 1:** Descriptive statistical analysis was conducted on 11 leaves phenotypic traits of 134 female trees from 13 natural populations of *Eucommia ulmoides* in China

Traits	Mean	SD	Min	Max	*CV*(%)
Leaf length (LL) (mm)	138.94	21.31	77.95	219.94	15.34
Petiole length (PL) (mm)	15.96	3.13	8.83	33.46	19.62
Length to width ratio of leaf (LTWR)	2.20	0.33	1.47	3.67	14.92
Leaf width (LW) (mm)	63.82	9.03	34.76	98.74	14.15
Leaf area (LA) (mm^2^)	5276.01	1410.18	1659.03	12599.59	26.73
Red component of leaf (RC)	71.77	8.36	52.00	127.00	11.64
Blue component of leaf (BC)	57.84	5.21	46.00	107.00	9.00
Green component of leaf (GC)	72.40	10.06	50.00	123.00	13.89
Chlorophyll reference value (CRV)	2.43	0.75	0.09	3.58	30.88
Leaf perimeter (LP) (mm)	386.09	57.40	206.94	605.10	14.87
Specific leaf dry weight (SLDW) (g/mm^2^)	1.00 × 10^−4^	0.21 × 10^−4^	0.38 × 10^−4^	1.74 × 10^−4^	20.94
Mean					17.45

### Overall phenotypic variation of *Eucommia ulmoides* leaves and differences in leaf traits among 13 natural populations

The nested analysis of variance results showed that the variation in the 11 leaf traits occurs in inter-populations and intra-populations (Table 2). Except for petiole length (PL) and length to width ratio of leaf (LTWR), which showed no significant differences in inter-populations, all other traits exhibited highly significant differences in both inter-populations and intra-populations (*p* < 0.01). Further multiple comparison analysis (Tables 3 and 4) indicated that the largest values for LA, maximum leaf width (LW), longest leaf length (LL), maximum leaf perimeter (LP), minimum LTWR, maximum SLDW, and highest CRV were observed in the population from Hunan. Among the populations, the Dayongqiao Sub-district (DYQ) population exhibited the widest leaves, with the highest values for LL (155.04 mm), LW (69.55 mm), LA (6242.52 mm^2^), and LP (436.23 mm). Conversely, the highest LTWR, shortest LL, smallest LP, and lightest leaf colour were observed in the population from Hubei. Specifically, the Hejia town (HJ) population had the shortest leaves (LL = 128.17 mm, LP = 346.56 mm, LA = 4512.37 mm^2^).

The variance components and phenotypic differentiation coefficients for the 11 leaf traits inter-populations and intra-populations were obtained through variance analysis (Table 2). The results indicate that the intra-population variance accounts for 46.58% of the total variance, while the inter-population variance accounts for 12.65% of the total variance. The phenotypic differentiation coefficient was 4.65-49.93%, with an average of 20.77%. Therefore, the leaf phenotypic diversity among the 13 natural populations of *E. ulmoides* is primarily attributed to intra-population variation. The trait with the highest phenotypic differentiation coefficient was GC, while the trait with the lowest coefficient was LTWR.

**Table 2:** Variance analysis and phenotypic differentiation coefficients of *Eucommia ulmoides* populations

Traits	Proportion of Variance Components (%)			Population Differentiation Coefficient (%)	F Value	
	Among Populations	Among Trees within Populations	Within Trees (Residual)		Among Populations	Among Trees within Populations
LL	8.10	49.22	42.69	14.13	2.63**	12.49***
PL	4.51	71.52	23.97	5.93	1.63	30.77***
LTWR	1.87	38.32	59.81	4.65	1.52	7.36***
LW	11.25	56.16	32.59	16.69	3.06**	18.15***
LA	12.03	57.33	30.63	17.35	3.20**	19.68***
RC	20.62	43.95	35.43	31.93	5.79***	13.43***
BC	14.23	31.68	54.09	30.99	5.08***	6.87***
GC	37.51	37.61	24.88	49.93	11.69***	16.11***
CRV	7.39	34.68	57.92	17.57	2.84**	6.94***
LP	9.29	54.11	36.60	14.66	2.77**	15.71***
SLDW	12.37	37.77	49.85	24.67	3.54***	8.51***
Mean	12.65	46.58	40.77	20.77		

* means *p* < 0.05; ** means *p* < 0.01; *** means *p* < 0.001. leaf length (LL), petiole length (PL), length to width ratio of leaf (LTWR), leaf width (LW), leaf area (LA), red component of leaf (RC), blue component of leaf (BC), green component of leaf (GC), chlorophyll reference value (CRV), leaf perimeter (LP), specific leaf dry weight (SLDW)

**Table 3:** Multiple comparison results for the 6 leaf traits in 13 *Eucommia ulmoides* populations

Population	PL	LL	LTWR	LW	LA	RC
ZX	17.43 ± 3.09 ab	139.64 ± 25.70 cde	2.22 ± 0.35 bcde	63.26 ± 8.13 c	5187.53 ± 1367.28 de	72.79 ± 6.63 c
QL	13.78 ± 2.52 f	130.93 ± 21.69 hi	2.10 ± 0.27 f	62.36 ± 7.18 c	4896.35 ± 1297.05 e	71.84 ± 5.98 c
MET	16.33 ± 2.82 cd	144.51 ± 15.71 bc	2.20 ± 0.30 cdef	66.58 ± 8.94 b	5631.59 ± 1160.17 bc	68.55 ± 5.72 d
DYQ	16.73 ± 2.80 bc	155.04 ± 22.49 a	2.25 ± 0.33 bcd	69.55 ± 8.18 a	6242.52 ± 1496.79 a	75.25 ± 6.41 b
JYLC	15.40 ± 2.93 de	147.20 ± 21.72 b	2.17 ± 0.25 def	68.00 ± 9.13 ab	5988.95 ± 1606.39 ab	71.70 ± 6.74 c
ZS	16.21 ± 2.73 cd	132.14 ± 25.92 ghi	2.12 ± 0.31 ef	62.40 ± 9.62 c	4906.61 ± 1608.03 e	75.81 ± 11.81 b
RS	16.16 ± 2.38 cd	137.99 ± 17.97 defg	2.10 ± 0.32 f	66.32 ± 7.73 b	5645.79 ± 1120.62 bc	78.65 ± 7.00 a
WF	17.64 ± 5.05 a	136.78 ± 20.13 efgh	2.38 ± 0.33 a	57.66 ± 5.10 d	4490.83 ± 795.50 f	69.48 ± 11.23 d
DGS	14.84 ± 1.76 e	133.66 ± 18.87 efghi	2.17 ± 0.40 def	62.83 ± 10.40 c	5203.73 ± 1289.59 de	68.59 ± 7.11 d
HJ	16.58 ± 3.11 bc	128.17 ± 17.75 i	2.16 ± 0.34 def	59.74 ± 6.14 d	4512.37 ± 946.89 f	62.89 ± 5.89 e
LH	14.68 ± 3.02 e	133.13 ± 17.50 fghi	2.28 ± 0.29 bc	58.86 ± 7.48 d	4461.43 ± 1061.84 f	72.91 ± 6.73 c
LA	15.93 ± 2.59 cd	143.43 ± 18.26 bcd	2.30 ± 0.39 ab	63.42 ± 10.38 c	5456.21 ± 1488.64 cd	75.02 ± 6.19 b
JY	16.67 ± 2.53 bc	139.33 ± 16.25 cdef	2.10 ± 0.29 f	67.03 ± 8.26 b	5619.59 ± 1170.27 bc	75.63 ± 6.68 b

Different letters in the same column indicate significant difference at the 0.05 level. petiole length (PL), leaf length (LL), length to width ratio of leaf (LTWR), leaf width (LW), leaf area (LA), red component of leaf (RC). Lianghe town, Gansu (LH); Wufeng town, Hubei (WF); Hejia town, Hubei (HJ); Donggongsi town, Guizhou (DGS); Zhongshan town, Guizhou (ZS); Jiangya forest farm, Hunan (JYLC); Jinyan town, Hunan (JY); Reshi town, Hunan (RS); Miaoertan town, Hunan (MET); Qianling town, Hunan (QL); Zhexi town, Hunan (ZX); Lean town, Hunan (LA); Dayongqiao Sub-district, Hunan (DYQ).

**Table 4:** Multiple comparison results for the 5 leaf traits in 13 *Eucommia ulmoides* populations

Population	BC	GC	CRV	LP	SLDW
ZX	59.37 ± 4.69 bc	72.31 ± 6.18 d	2.61 ± 0.26 abc	390.88 ± 63.28 bcd	1.11 × 10^−4^ ± 0.21 × 10^−4^ a
QL	56.35 ± 2.47 ef	72.15 ± 5.42 d	2.69 ± 0.19 ab	372.49 ± 59.54 ef	1.00 × 10^−4^ ± 0.18 × 10^−4^ cde
MET	55.39 ± 3.37 fg	68.65 ± 5.77 e	2.81 ± 0.23 a	396.49 ± 47.85 bc	0.99 × 10^−4^ ± 0.18 × 10^−4^ cdef
DYQ	60.24 ± 3.86 b	77.67 ± 5.72 b	2.44 ± 0.23 cdef	436.23 ± 50.33 a	0.94 × 10^−4^ ± 0.18 × 10^−4^ f
JYLC	57.07 ± 3.30 de	75.32 ± 7.16 c	2.53 ± 0.43 bcd	405.39 ± 63.06 b	0.98 × 10^−4^ ± 0.17 × 10^−4^ def
ZS	59.25 ± 8.07 bc	74.12 ± 12.52 cd	1.91 ± 1.18 h	376.17 ± 68.33 def	0.76 × 10^−4^ ± 0.22 × 10^−4^ g
RS	61.70 ± 5.86 a	82.02 ± 6.80 a	2.30 ± 0.28 ef	382.39 ± 41.60 cdef	1.10 × 10^−4^ ± 0.18 × 10^−4^ a
WF	56.17 ± 5.57 ef	68.88 ± 14.11 e	2.23 ± 1.06 fg	365.86 ± 45.97 f	1.04 × 10^−4^ ± 0.24 × 10^−4^ bcd
DGS	57.90 ± 6.70 cd	65.82 ± 7.62 f	2.68 ± 0.69 ab	378.73 ± 53.71 def	0.96 × 10^−4^ ± 0.23 × 10^−4^ ef
HJ	54.11 ± 5.17 g	58.36 ± 5.77 g	2.05 ± 1.46 gh	346.56 ± 41.63 g	1.08 × 10^−4^ ± 0.18 × 10^−4^ ab
LH	58.61 ± 4.92 c	73.14 ± 6.84 cd	2.41 ± 0.74 cdef	386.73 ± 55.48 cde	0.96 × 10^−4^ ± 0.19 × 10^−4^ ef
LA	58.97 ± 3.08 bc	77.82 ± 6.29 b	2.47 ± 0.23 bcde	389.52 ± 50.10 bcde	0.98 × 10^−4^ ± 0.22 × 10^−4^ ef
JY	60.26 ± 3.75 b	80.34 ± 7.06 a	2.36 ± 0.26 def	391.38 ± 49.11 bcd	1.05 × 10^−4^ ± 0.16 × 10^−4^ bc

Different letters in the same column indicate significant difference at the 0.05 level. blue component of leaf (BC), green component of leaf (GC), chlorophyll reference value (CRV), leaf perimeter (LP), specific leaf dry weight (SLDW). The full names corresponding to population abbreviations are the same as those in Table 3.

### Principal component analysis of the 11 leaf traits

**Fig. 2:**
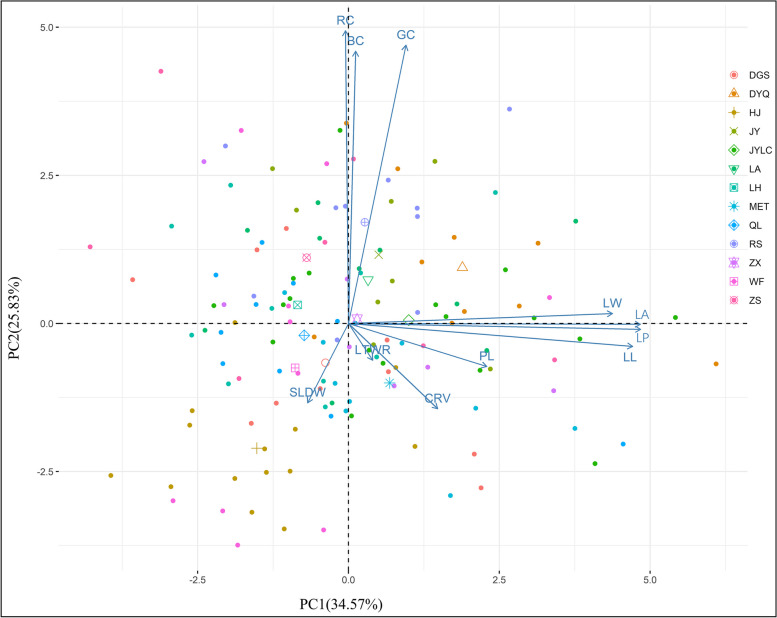
Biplot of the principal components analysis for the 11 leaf traits

 The principal component analysis (PCA) results indicate that the first three principal components have eigenvalues greater than 1, and together, they account for 74.98% of the total variation in the EULs traits (Table 5). This suggests that the first three principal components can explain a significant portion of the variation in the EULs traits. For the first principal component, traits such as LP (0.958), LA (0.957), LL (0.932), and LW (0.866) had relatively large positive eigenvectors. These traits are primarily associated with leaf size. For the second principal component, the positive eigenvectors for RC, GC, and BC were all higher than 0.9. These traits are primarily associated with leaf colour. For the third principal component, the positive eigenvector for the LTWR was the largest, indicating that it primarily reflected leaf shape. As shown in Fig. 2, the vector lengths of the means of the Reshi town, Hunan (RS), Jinyan town, Hunan (JY), Lean town, Hunan (LA), Zhexi town, Hunan (ZX), Jiangya forest farm, Hunan (JYLC), and Dayongqiao Sub-district, Hunan (DYQ) populations along the PC1 axis were relatively large. Among the populations, the DYQ population had the longest vector along the PC1 axis.

**Table 5:** Principal component analysis of the 11 leaf traits

Traits	PC1	PC2	PC3
LL	0.932	-0.077	0.284
PL	0.453	-0.144	0.521
LTWR	0.079	-0.123	0.912
LW	0.866	0.033	-0.461
LA	0.957	-0.002	-0.214
RC	-0.010	0.978	0.074
BC	0.024	0.909	0.217
GC	0.188	0.929	0.067
CRV	0.292	-0.285	0.087
LP	0.958	-0.020	0.031
SLDW	-0.134	-0.265	0.313
Eigen value	3.803	2.841	1.604
Contribution rate	34.573	25.825	14.585
Cumulative Contribution rate	34.573	60.399	74.984

leaf length (LL), petiole length (PL), length to width ratio of leaf (LTWR), leaf width (LW), leaf area (LA), red component of leaf (RC), blue component of leaf (BC), green component of leaf (GC), chlorophyll reference value (CRV), leaf perimeter (LP), specific leaf dry weight (SLDW)

### Correlations between leaf phenotypic traits

**Fig. 3:**
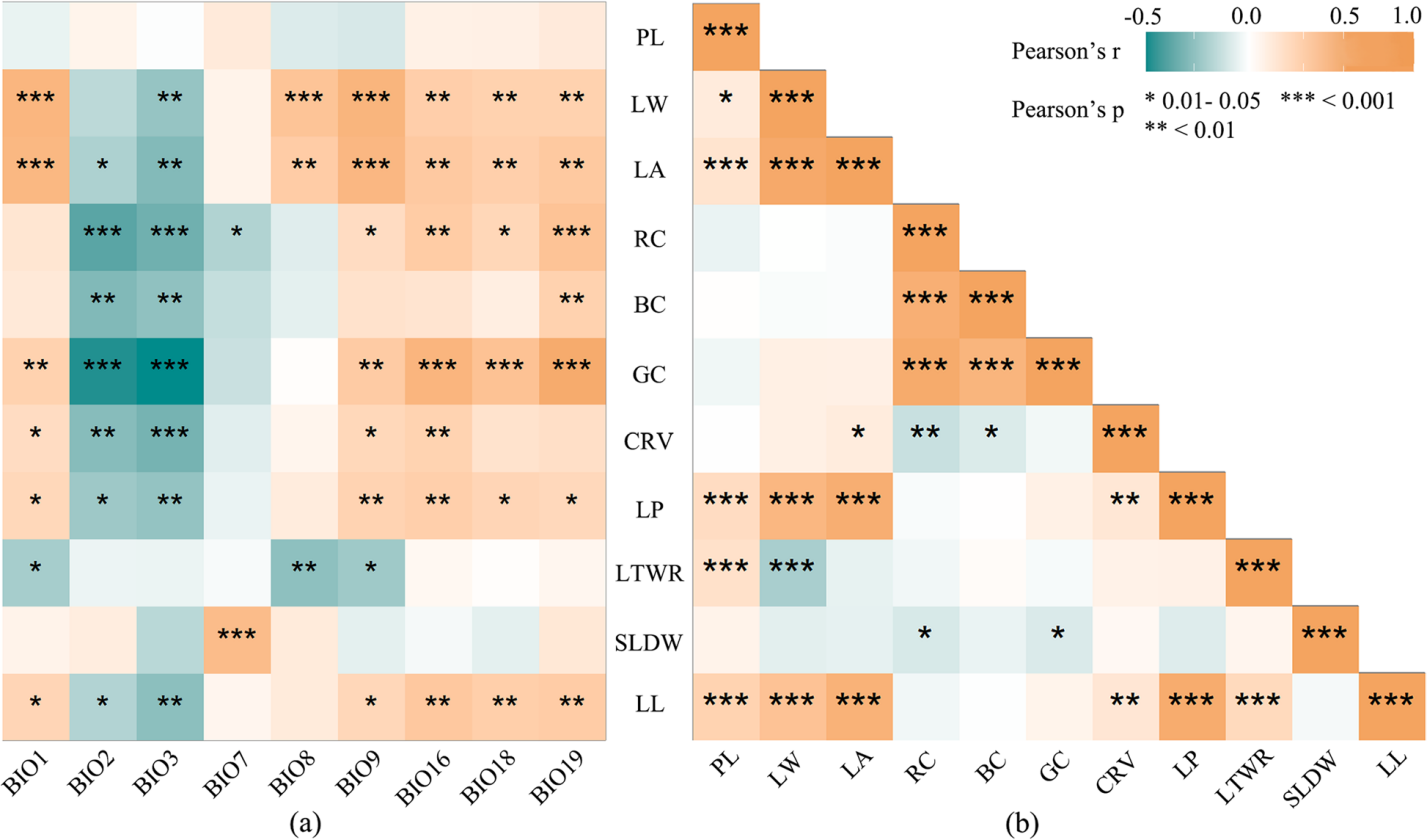
Correlation between leaf traits and correlation between leaf traits and climate. **a** Correlations between 11 leaf traits and climatic factors. **b** Correlations among leaf traits. Green color represents negative correlation, orange color represents positive correlation, and the darker the color, the stronger the correlation

 The correlation analysis of the 11 leaf traits (Fig. 3b) revealed that out of the 55 pairs of relationships examined, 18 pairs exhibited a very significant correlation (*p* ≤ 0.01), while 5 pairs showed a significant correlation (*p* ≤ 0.05). Among them, 6 traits related to leaf shape and size (LTWR, LL, LP, LW, LA, and PL), as well as 3 traits related to leaf colour (GC, RC, and BC), exhibited an extremely significant positive correlation. PL and LW show a significant positive correlation. LW and LTWR exhibited a highly significant negative correlation. SLDW was significantly negatively correlated with BC and GC.

### Effects of geographical factors on the phenotypic traits of leaves

The mantel test results indicated a significant correlation between altitude and leaf traits (*p* < 0.05), while latitude and longitude did not show significant correlations with the traits (Table 6). The correlation analysis revealed a significant positive correlation between longitude and various traits (LW, LA, SLDW, and LL). Latitude was significantly negatively correlated with LW, LA, RC, BC, and GC and significantly positively correlated with SLDW. Altitude exhibited a significant negative correlation with LW, LA, LP, LL, SLDW, and GC (Tables 7 and 8). Both the mantel test and correlation analysis results showed the predominant influence of altitude on *Eucommia* leaf traits.

Geographical distance showed a significant correlation with climatic distance (*r* = 0.78, *p* = 0.001). After controlling for climatic factors, some significant relationships between leaf traits and geographical factors disappeared (Tables 7 and 8). Regarding the relationships between latitude, longitude, and SLDW, a significant correlation persisted after controlling for the influence of precipitation. In the case of the relationships between altitude and leaf traits, after controlling for climatic factors, it was found that the significant correlations of SLDW, LL, and GC disappeared. However, after controlling for the influence of precipitation, a significant negative correlation with LW and LA was still observed. Furthermore, after controlling for the influence of temperature, a significant negative correlation with LP still existed.

**Table 6:** Mantel test analyzes the influence of geography, climate, latitude, longitude and altitude distance on the phenotypic distance of *Eucommia ulmoides* leaves

	Climatic distance	Geographical distance	Longitude distance	Latitude distance	Altitude distance
Leaf phenotypic distance	*r* = 0.51, *p* = 0.004	*r* = 0.33, *p* = 0.048	*r* = 0.02, *p* = 0.435	*r* = 0.28, *p* = 0.074	*r* = 0.32, *p* = 0.021

**Table 7:** Correlation coefficient between 6 leaf traits and geographical factors after controlling climate factors

	PL	LW	LA	LP	LTWR	SLDW
LON	0.169	0.175*	0.199*	0.057	0.011	0.322**
LON (Control 1)	0.182	0.011	0.018	-0.084	0.016	-0.030
LON (Control 2)	0.095	-0.010	-0.036	-0.109	-0.017	0.306**
LON (Control 1 and 2)	-0.097	-0.110	-0.180	-0.266	-0.175	0.020
LAT	0.008	-0.217*	-0.240**	-0.167	0.060	0.197*
LAT (Control 1)	-0.183	-0.024	-0.047	0.066	-0.022	-0.007
LAT (Control 2)	0.085	-0.062	-0.066	0.006	0.126	0.284**
LAT (Control 1 and 2)	-0.106	-0.034	-0.070	-0.055	-0.018	-0.153
AL	-0.007	-0.357**	-0.354**	-0.204*	0.141	-0.207*
AL (Control 1)	0.059	-0.087	-0.060	-0.187*	0.010	0.097
AL (Control 2)	0.095	-0.305**	-0.262**	-0.141	0.223	-0.131
AL (Control 1 and 2)	0.097	-0.104	-0.064	-0.124	0.086	0.144

** means *p* < 0.01, * means *p* < 0.05, LON: longitude, LAT: latitude, ALT: altitude, 1: temperature-related factors (BIO1, BIO2, BIO3, BIO7, BIO8, BIO9), 2: Precipitation related factors (BIO16, BIO18, BIO19), petiole length (PL), leaf width (LW), leaf area (LA), leaf perimeter (LP), length to width ratio of leaf (LTWR), specific leaf dry weight (SLDW)

**Table 8:** Correlation coefficient between 5 leaf traits and geographical factors after controlling climate factors

	LL	RC	BC	GC	CRV
LON	0.186*	0.029	-0.005	0.150	0.018
LON (Control 1)	0.030	-0.068	-0.140	-0.082	-0.151
LON (Control 2)	-0.007	-0.373	-0.363	-0.371	-0.036
LON (Control 1 and 2)	-0.241	-0.100	-0.218	-0.150	-0.023
LAT	-0.155	-0.296**	-0.240**	-0.326**	-0.168
LAT (Control 1)	-0.033	-0.040	-0.006	-0.043	0.135
LAT (Control 2)	0.066	-0.151	-0.191	-0.077	-0.009
LAT (Control 1 and 2)	-0.052	0.074	0.021	0.046	-0.110
ALT	-0.242**	-0.108	-0.099	-0.250**	-0.156
ALT (Control 1)	-0.094	0.136	0.087	0.097	-0.118
ALT (Control 2)	-0.128	0.123	0.091	0.035	-0.129
ALT (Control 1 and 2)	-0.026	0.017	-0.039	-0.042	-0.052

** means *p* < 0.01, * means *p* < 0.05, LON: longitude, LAT: latitude, ALT: altitude, 1: temperature-related factors (BIO1, BIO2, BIO3, BIO7, BIO8, BIO9), 2: Precipitation related factors (BIO16, BIO18, BIO19), leaf length (LL)red component of leaf (RC), blue component of leaf (BC), green component of leaf (GC), chlorophyll reference value (CRV)

### Effects of climatic factors on leaf phenotypic traits

The mantel test results indicated significant correlations between climate and leaf traits (*p* < 0.01) (Table 6). The correlation analysis revealed that temperature and precipitation had a significant impact on leaf traits (Fig. 3a). For instance, LA, LP, LL, and LW decreased with an increase in mean diurnal range (BIO2) and isothermality (BIO3) but increased with an increase in annual mean temperature (BIO1), mean temperature of wettest quarter (BIO8), mean temperature of driest quarter (BIO9), precipitation of wettest quarter (BIO16), precipitation of warmest quarter (BIO18), and precipitation of coldest quarter (BIO19). RC and GC were positively correlated with BIO9, BIO16, BIO18, and BIO19. RC, GC, and BC were negatively correlated with BIO2 and BIO3. SLDW was positively correlated with temperature annual range (BIO7). Among individual climate factors, seven variables explained a significant portion of the leaf trait variation: BIO1, BIO2, BIO16, and BIO19 explained variation in seven leaf traits, while BIO3 and BIO9 explained variation in eight leaf traits.

## Discussion

### Leaf phenotypic variation

Studying the correlations between various leaf traits is beneficial for understanding the adaptive strategies of leaves in different environments [35]. In this study, traits characterizing leaf size, including LL, LTWR, LP, LA, LW, and PL, showed a significant positive correlation (Fig. 3b). As leaves grow larger, plants need to acquire more light energy, hence requiring longer petioles to reduce mutual shading within the individual [36]. However, both LW and LTWR exhibited a highly significant negative correlation while SLDW showed a significant negative correlation with BC and GC. These negative correlations reflect the trade-off strategies employed by plants in different environments [37].

The analysis of variation in eleven leaf phenotypic traits of *E. ulmoides* revealed coefficients of variation (*CV*) ranging from 9.00 to 30.88%, with an average of 17.45% (Table 1). These values are similar to those found in studies on *Acer mono* Maxim (18.07%) [38] and *Davidiain volucrata* (16.22%) [39]. Notably, in this study, we observed the highest *CV* for the CRV (30.88%), followed by leaf area (26.73%). This differs from the findings of Meng et al., where leaf area exhibited the highest *CV* (20.68%) and CRV showed the lowest *CV* (4.57%) [31]. Chlorophyll is a green pigment in plants, and its content directly influences photosynthesis [40]. The concentration of chlorophyll is primarily influenced by atmospheric and soil factors [41]. This variation in the CRV may be attributed to the more diverse natural habitats of the *E. ulmoides* populations studied here compared to those in the germplasm resource base. This increased habitat diversity likely contributed to the observed higher variation in the CRV.

In this study, rich variation was observed in traits representing leaf size (Table 1), which aligns with previous findings on leaf variation within natural populations of *E. ulmoides* [32]. Leaf size directly influences a plant’s ability to capture light and acquire carbon [42], and it can also reflect the content of active components within the leaf [8, 9]. Additionally, morphological scans of *E. ulmoides* leaves revealed a diverse range of variations in leaf quality traits, particularly the leaf base, the leaf tip, and leaf shape (Fig. 1). Among these, leaf shape exhibits the highest degree of variation, encompassing eight distinct types, including lanceolate, ovate, elliptic, and inverse lanceolate forms and others. Changes in leaf shape provide a visual representation of the plant’s adaptation to environmental shifts; for instance, oval-shaped leaves exhibit higher photosynthetic efficiency and water use efficiency than lanceolate leaves [6, 7]. As *E. ulmoides* serves both medicinal and dietary purposes, further research is needed to ascertain whether leaf shape and size can serve as indicators of the content of active components within EULs.

### Sources of leaf phenotypic variation

The 11 leaf phenotypic traits of *E. ulmoides* exhibit significant variations both intra- and inter-populations (Table 2), which is consistent with findings for *Litsea coreana* Levl. var. *sinensis* [10] and *Carpinus tschonoskii* [43]. The average coefficient of variation for leaf phenotypic traits across the 13 *E. ulmoides* populations was 20.77%, which is lower than that of *Tetracentron sinense* (46.69%) [44] and *Phoebe chekiangensis* (Lauraceae) (41.43%) [45] but higher than that of *Azadirachta indica* (11.89%) [46]. In comparison with these other woody plants, *E. ulmoides* populations show a moderate degree of leaf phenotypic variation, with most attributable to intra-population variation. Hamrick et al. suggested that outcrossing plants can mitigate the impact of genetic drift on genetic structure, facilitating the maintenance of low levels of genetic differentiation inter-populations [47]. *E. ulmoides* is a dioecious plant. From the 1950s to the late 1980s, traditional bark harvesting for medicinal purposes led to severe over-exploitation of *E. ulmoides* resources, resulting in their current scarcity and fragmented distribution [48]. Consequently, cross-pollination between populations of *E. ulmoides* is relatively challenging, thereby providing conditions conducive to genetic differentiation inter-populations.

Under natural conditions, *E. ulmoides* seedlings take 7–8 years to flower, resulting in limited seed production [49]. *E. ulmoides* seeds face persistent challenges such as low germination rates, poor seedling development, and a loss of germination capacity in the second year [50]. This leads to slow self-renewal intra-populations, promoting genetic differentiation intra-populations. Field investigations have revealed that *E. ulmoides* populations are widely distributed, primarily in mountainous areas, which increases the difficulty of inter-population pollination. Additionally, *E. ulmoides* fruits are relatively heavy (with a weight of 4.24–13.42 g per hundred seeds) [32], making long-distance seed dispersal challenging. Some populations show high plant density, intensifying genetic exchange intra-populations and thereby increasing the level of differentiation intra-populations. Consequently, intra-population variation serves as the primary source of leaf variation in *E. ulmoides* populations.

### Relationship between leaf phenotype and environmental factors

In this study, the mantel test results revealed significant correlations between altitude and leaf traits (Table 6). Traits representing leaf size, such as LW, LA, and LP, decreased with increasing altitude (Tables 7 and 8), consistent with *Ternstroemia lineata* [14] and *Salix triandra* L. [51]. In high-altitude, low-temperature environments, smaller leaves incur lower respiration and transpiration costs, reducing the plant’s maintenance expenditure [52]. Additionally, at higher altitudes, regions experience higher wind speeds, and smaller leaves are more wind-resistant than larger leaves [22]. This aligns with the results of the PCA (Fig. 2), where populations in lower-altitude regions (RS, JY, LA, ZX, JYLC, and DYQ) had larger leaves than populations in higher-altitude regions (ZS, LH, WF, DGS, HJ, QL), with plants in DYQ exhibiting the largest leaves. The results of multiple analyses also corroborate this conclusion (Tables 3 and 4). Furthermore, latitude and longitude show no significant correlation with the traits, indicating that the 11 leaf phenotypic traits do not exhibit a consistent geographic variation pattern along latitudinal and longitudinal gradients.

Climate has a significant impact on the leaf phenotypic traits (*p* < 0.01). LA, LP, LL, and LW are positively correlated with BIO1, BIO8, BIO9, BIO16, BIO18, and BIO19, indicating that in regions with higher temperatures and greater precipitation, leaves tend to be larger (Fig. 3a). This finding is consistent with those for *Litsea coreana* var. *sinensis* [10] and *Tetracentron sinense* Oliv [15]. Larger leaves have thicker boundary layers, which slow sensible heat exchange with the surrounding air. [16]. All leaves cool themselves through transpirational water loss. When water supply is insufficient, plants reduce leaf area to minimize water consumption and prevent the leaf surface from becoming excessively hot [18, 53]. Therefore, in regions with lower altitudes, higher temperatures, and higher precipitation, the leaves of *E. ulmoides* tend to be larger.

### Protection and management strategy

Based on the results of this study and considering the current shortage of *E. ulmoides* resources, the following conservation and management strategies are proposed: (1) Prioritize the protection of high-quality germplasm resources. Using high coefficients of variation and low phenotypic differentiation coefficients as selection criteria for desirable traits is more reliable than other approaches. Additionally, high-quality germplasm resources may be more prevalent in regions with lower altitudes, higher temperatures, and lower precipitation. This could lead to a modification of sampling strategies. (2) *E. ulmoides* is an endemic monotypic tree species in China with a wide range of adaptability and clear geographical advantages. As the population distribution gradually shrinks, establishing germplasm resource protection through individual transplants is recommended to facilitate gene exchange. (3) Reducing human activities is a crucial measure for strengthening the protection of existing resources and habitats.

## Conclusions

The leaf phenotypic variation in different populations of *E. ulmoides* is abundant, with a diverse range of morphological variations, including in leaf shape. Significant differences in the variation in 11 leaf phenotypic traits existed both in intra- and inter-populations, exhibiting a gradient pattern with respect to altitude. Regarding climatic factors, the leaf phenotypic variation is closely associated with local climate variables such as temperature and precipitation at the sampling sites. The variability in *E. ulmoides* leaf traits primarily arises from intra-population variation. In the future, the collection of germplasm resources should focus on selecting representative individual samples from populations in low-altitude and warm regions. In summary, *E. ulmoides* exhibits rich leaf phenotypic variation, showing strong adaptability to different environments. This variation is conducive to expanding its range of adaptation and supports the breeding, utilization, and evaluation of *E. ulmoides* germplasm resources.

## Materials and methods

### Plant materials

**Fig. 4:**
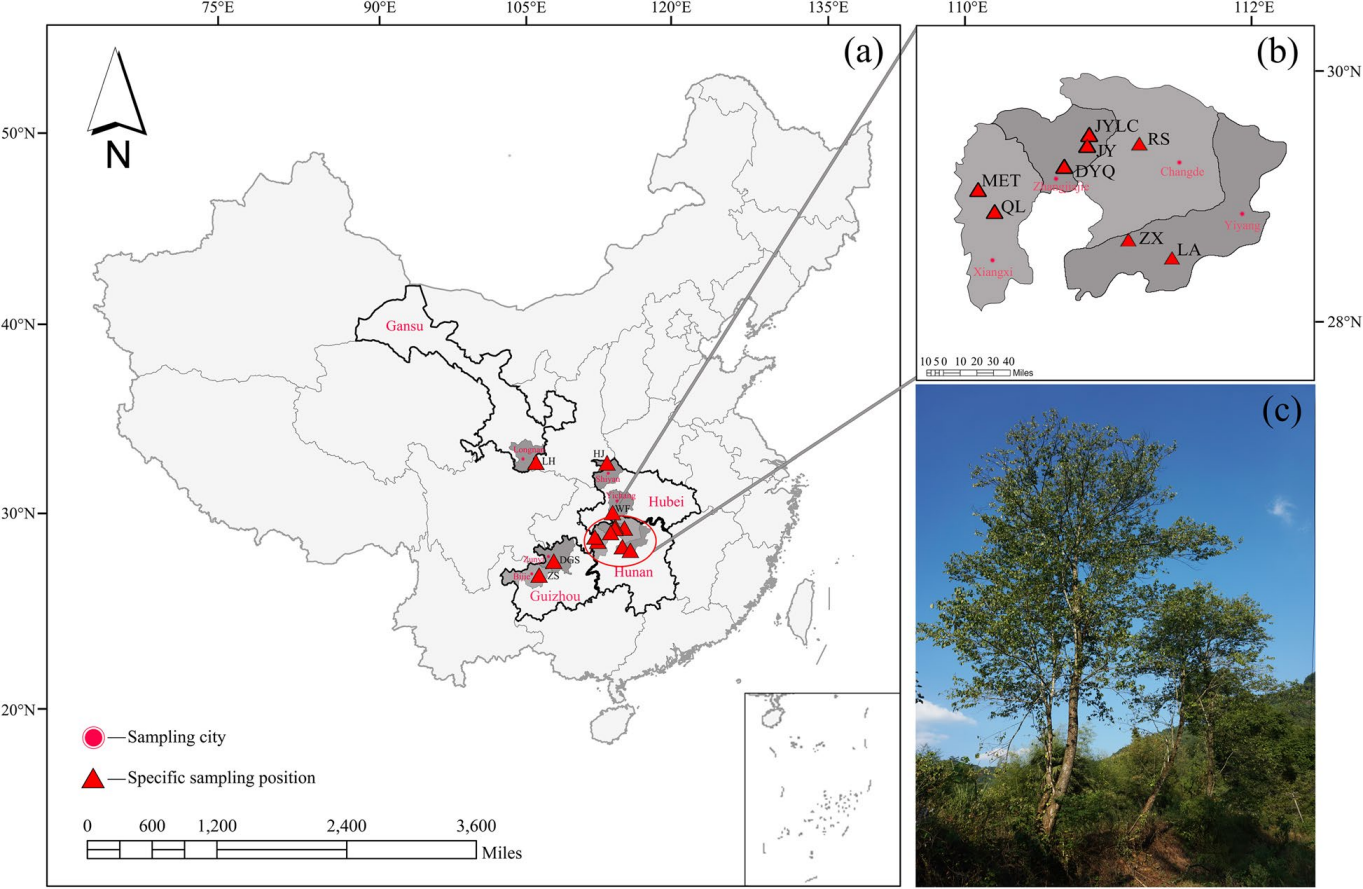
*Eucommia ulmoides* (*E. ulmoides*) sampling map. **a** The gray area in the figure represents the geographical range where the sampling sites of the 13 natural populations of *E. ulmoides* are located, **b** Enlarged figure of sampling sites of 8 natural populations of *E. ulmoides* in Hunan Province, **c** Adult tree of *E. ulmoides* natural population. The specific locations are as follows: Lianghe town, Gansu (LH); Wufeng town, Hubei (WF); Hejia town, Hubei (HJ); Donggongsi town, Guizhou (DGS); Zhongshan town, Guizhou (ZS); Jiangya forest farm, Hunan (JYLC); Jinyan town, Hunan (JY); Reshi town, Hunan (RS); Miaoertan town, Hunan (MET); Qianling town, Hunan (QL); Zhexi town, Hunan (ZX); Lean town, Hunan (LA); Dayongqiao Sub-district, Hunan (DYQ). (The maps are created by authors using ArcGIS software)

 In September and October 2022, a total of 134 female tree samples were selected from 13 natural populations. The basic information on the populations and sampling sites can be found in Fig. 4; Table 9. Within each population, the distance between sampled trees was set to 30 m to reduce their relatedness. For each tree, 10 fresh and mature leaves without obvious diseases or pests were collected from the middle branches in four directions (east, south, west, and north). These leaves were used for measuring morphological traits [54].

**Table 9:** Population, number of trees sampled and geographic factors for 13 natural populations *Eucommia ulmoides*

Population	Number of	Longitude (°E, LON)	Latitude (°N, LAT)	Altitude (m, ALT)
LH (Lianghe town, Gansu)	10	105.88	33.17	1073-1098
WF (Wufeng town, Hubei)	10	110.58	30.09	930–1430
HJ (Hejia town, Hubei)	14	110.60	32.94	420–780
DGS (Donggongsi town, Guizhou)	10	106.88	27.73	924–978
ZS (Zhongshan town, Guizhou)	7	106.03	27.00	1221-1389
JYLC (Jiangya forest farm, Hunan)	18	110.77	29.52	140–360
JY (Jinyan town, Hunan)	8	110.71	29.14	210-610
RS (Reshi town, Hunan)	10	111.25	29.37	250–280
MET (Miaoertan town, Hunan)	10	109.48	28.87	690–720
QL (Qianling town, Hunan)	10	109.68	28.80	470–570
ZX (Zhexi town, Hunan)	7	111.17	28.34	110–280
LA (Lean town, Hunan)	10	111.60	28.10	208–387
DYQ (Dayongqiao Sub-district, Hunan)	10	110.46	29.14	216–232

### Collection of environmental data

Geographical data (longitude, latitude, and altitude) of the populations were obtained using GPS 315 (Magellan). Climate data were obtained from WorldClim v2.1 (http://www.worldclim.org/), which provides global meteorological data in raster format [55]. A total of 19 meteorological variables were extracted for the corresponding populations using ArcGIS 10.8. Correlation analysis was performed to remove strongly correlated factors (*r*^*2*^ ≥ 0.9) among the meteorological variables [56]. Finally, 9 climatic factors and 3 geographic factors were selected, and the results are shown in Table 10.

**Table 10:** Climatic and geographic factors

**Name**	**Description**	**Name**	**Description**
LON (°E)	Longitude	BIO7 (°C)	Temperature annual range
LAT (°N)	Latitude	BIO8 (°C)	Mean temperature of wettest quarter
ALT (m)	Altitude	BIO9 (°C)	Mean temperature of driest quarter
BIO1 (°C)	Annual mean temperature	BIO16 (mm)	Precipitation of wettest quarter
BIO2 (°C)	Mean diurnal range	BIO18 (mm)	Precipitation of warmest quarter
BIO3	Isothermality	BIO19 (mm)	Precipitation of coldest quarter

### Measurement of leaf phenotype

10 leaves were selected from each tree to measure their 11 phenotypic traits. The LA-S Plant Leaf Image Analyzer (Hangzhou Wanshen Detection Technology Co., Ltd.) was used to measure the following leaf phenotypic traits: LL, LW, LA, RC, BC, GC, CRV, and LP. RGB is a color model that describes colors using the intensities of red, green, and blue primary colors. In this study, the RGB values were used to describe the color characteristics of the leaves [31]. The PL was measured using a vernier caliper. The SLDW is the ratio of leaf dry mass to leaf area. According to the Chinese Pharmacopoeia (2020 edition), fresh leaves were dried to constant weight at low temperature, and then the dry weight of leaves was measured by one-thousandth electronic balance.

### Method statement

The investigation and collection of *E. ulmoides* leaf samples in this study have been approved by the local regulatory authorities. The mature leaves of *E. ulmoides* were identified by Professor Boru Liao from Jishou university as belonging to the genus *Eucommia* in the family *Eucommiaceae*. A voucher specimen has been deposited in the Herbarium of the College of Biology and Environmental Sciences, Jishou university, with voucher number JSU-EU116.

### Statistical analysis

Statistical analysis was performed to calculate the maximum, minimum, mean ($\stackrel{-}{X}$), standard deviation (SD), and coefficient of variation (*CV*) for all 11 phenotypic traits of the leaves. *CV* was calculated as:

$$CV=(SD/\stackrel{-}{X})\times 100{\%}$$

The linear model used for conducting nested analysis of variance for leaf traits is as follows:

$${Y}_{ijn}={\upmu }+{\alpha }_{i}+{\beta }_{j\left(i\right)}+{\epsilon }_{\left(ij\right)n}$$

where ${\upmu }$ is the overall average, ${\alpha }_{i}$ is the random effective value of the *i*th population, ${\beta }_{j\left(i\right)}$ is the random effective value of the *j*th tree in the *i*th population and ${\epsilon }_{\left(ij\right)n}$ is the experimental error of the *ijn*th observation value, which is the variation within trees [57].

The formula for calculating the population differentiation coefficient is:

$${V}_{st}=\frac{{\sigma }_{i}^{2}}{{\sigma }_{i}^{2}+{\sigma }_{j\left(i\right)}^{2}}\times 100{\%}$$

where ${\sigma }_{i}^{2}$ is the variance among populations and ${\sigma }_{j\left(i\right)}^{2}$ is the variance within the population.

After the nested analysis of variance, multiple comparison analysis was conducted using the Duncan method to compare the specific differences in leaf traits inter-populations [58]. Pearson correlation analysis was used to investigate the correlation between leaf phenotypic traits and the influence of climate and geographical factors on leaf trait variation. After controlling climate factors by partial correlation analysis, the correlation between geography and leaf traits was studied. The data were standardized by Z-sore, and the PCA was carried out after the influence of dimensions was eliminated. PCA was used to condense phenotypic traits into several principal components and explore the structure and relationships of leaf traits inter-populations. The data statistics were analyzed using Excel 2016, SPSS 26.0, and R 4.1.3 software.

## Data Availability

The data that support the findings of this study are available from the corresponding author upon reasonable request.
